# Etymologia

**DOI:** 10.3201/eid1706.ET1706

**Published:** 2011-06

**Keywords:** etymologia, yaws

## Yaws [yôz]

From either the Carib *yaya*, for sore or lesion, or *yaw*, an African word for berry. The term yaws was in common use by the 17th Century, when Dutch physician Willem Piso provided one of the earliest recorded descriptions of yaws in South America in De medicina Brasiliense in 1648. Because lesions associated with the disease resemble berries, another common name for yaws is frambesia tropica, from the French *framboise*, meaning raspberry.

